# Medical College and Hospital Notes

**Published:** 1896-04

**Authors:** 


### Medical College and Hospital Notes.

---

THE directors of the Post-Graduate Medical School and Hospital have named one of their wards in memory of the late Dr. Charles Carroll Lee, who was for many years a professor in the institution. They have placed a tablet in the ward, giving the names of those who combined to contribute the $10,000 which was given for the purpose of the memorial. These names are as follows: Dr. Robert Abbe, Dr. L. Bolton Bangs, Mrs. James Beales, Dr. Stephen S. Burt, Miss Caldwell, Dr. Charles L. Dana, Dr. Bache McE. Emmet, Dr. George H. Fox, "A Friend," Dr. Horace T. Hanks, Mr. and Mrs. Eugene Kelly, Mr. and Mrs. Henry J. Lamarche, Dr. Daniel Lewis, Mr. and Mrs. William Lummis, Mr. and Mrs. Frank A. Otis, Dr. Clarence C. Rice, Mr. Eli K. Robinson, Mr. Nelson Robinson, Dr. D. B. St. John Roosa, Mrs. Eliza M. Sloan, Dr. Andrew H. Smith, Mrs. M. E. Sparks and Dr. Reynold W. Wilcox. It will be seen that the faculty of the institution participated largely in the memorial gift.

---

THE Buffalo Hospital of the Sisters of Charity will formally open the new wing that has recently been constructed, on Saturday, April 11, 1896. A reception will be held, lasting from 3 to 9 P. M., at which a committee of women, belonging to the St. Elizabeth Hospital Association, will receive the guests. Refreshments will be served and the new building will be open to visitors during the next day, Sunday, April 12th. A number of people have promised to furnish private rooms, among whom is Mrs. Edwin G. S. Miller. There are still a number of private rooms and ward beds to be furnished, and the managers hope that all who have promised will fulfil in time for the opening.

---

The Riverside Hospital, of Buffalo, has issued a very tasteful circular, announcing that it is conducted for the medical and surgical treatment of men, women and children. It was established by Dr. Lillian Craig Randall, who is the physician in charge. Dr. Randall is aided by a staff of prominent attending and consulting physicians.

A training class for nurses is established in connection with the hospital, where pupils are received from May 1st. Further particulars may be obtained by addressing Dr. Lillian Craig Randall, 327 Breckenridge street, Buffalo.

---

The faculty of the medical department of the University of Buffalo has issued a circular, inviting the profession to a series of lectures on comparative pathology by Dr. Woods Hutchinson, who has long given attention to the subject. The course began on Tuesday, March 17, and will close on Thursday, April 2, 1896. The lectures are given at 5 o'clock P. M., in Alumni Hall, at the University building.

---

Official.—The medical department of the University of Buffalo makes the official announcement to the profession and to students of medicine that it has adopted a four years' course, beginning with the session of 1896–'97. All matriculants after March 5, 1896, will be required to comply with this regulation.

